# Mechanism of HBx carcinogenesis interaction with non-coding RNA in hepatocellular carcinoma

**DOI:** 10.3389/fonc.2023.1249198

**Published:** 2023-09-08

**Authors:** Zhuoran Wang, Nan Li, Peng Cai, Cunzhen Zhang, Guangwen Cao, Jianhua Yin

**Affiliations:** ^1^ Department of Hepatic Surgery I (Ward I), Shanghai Eastern Hepatobiliary Surgery Hospital, Navy Medical University, Shanghai, China; ^2^ Department of Epidemiology, Faculty of Navy Medicine, Navy Medical University, Shanghai, China

**Keywords:** non-coding RNA, HBx, hepatitis B, hepatocellular carcinoma, interaction

## Abstract

Hepatocellular carcinoma (HCC) is an extremely malignant tumor that affects individuals throughout the world. One of the main causes of HCC is hepatitis B virus (HBV). Therefore, it is crucial to understand the mechanisms underlying HBV carcinogenesis. Increasing evidence suggests that the HBV X protein (HBx), which is encoded by HBV, plays a significant role in cell apoptosis, DNA damage repair, and cell cycle regulation. This ultimately leads to the development of HCC. Additionally, recent studies have shown that non-coding RNA (ncRNA) also contributes to the carcinogenesis and pathogenesis of different of tumors. ncRNA plays a significant role in the formation of HCC by regulating the inflammatory signaling pathway, activating immune cells, and modifying epigenetics. However, it remains unclear whether ncRNA is involved in the regulation of the carcinogenic mechanisms of HBx. This article reviews the carcinogenic mechanism of HBx and its interaction with ncRNA, providing a novel strategy for the clinical diagnosis and treatment of HCC.

## Introduction

Liver cancer ranks as the sixth most common cancer worldwide and is the third leading cause of cancer-related deaths. There are approximately 906,000 new cases and 830,000 deaths attributed to liver cancer every year. Hepatocellular carcinoma (HCC), the most common pathological subtype of liver cancer, has the highest incidence and mortality rates in Asia, accounting for 75% to 85% of cases (1). Despite ongoing improvements in the diagnosis and treatment of HCC, early clinical symptoms are often not apparent, leading to a delayed diagnosis for patients. Furthermore, the characteristics of HCC, such as high rates of recurrence and metastasis, continue to present challenges for effective treatment (2). Hepatitis B virus (HBV) infection is the leading cause of HCC in China (3). Despite extensive research, the pathogenesis of HBV infection is not yet fully understood. Previous studies have shown that integration of the HBV genome into the host genome can lead to gene mutations (4) or epigenetic changes in the host (5). Furthermore, the HBx protein and the HBV S protein, encoded by HBV, are involved in the regulation of various signaling pathways, increasing the susceptibility to cancer predisposing factors (6, 7), and promoting the appearance and development of HCC (8). As an important viral protein with multiple carcinogenic functions encoded by HBV, the HBx protein plays an essential role in the occurrence and development of HCC, including the regulation of viral replication and interference with multiple key signaling pathways related to tumorigenesis (9), and the regulation of the expression of different genes and proteins that influence cell proliferation, mitochondrial activity, DNA damage repair, and other functional activities (10). Non-coding RNA (ncRNA) also plays an important role in the occurrence and development of HCC by regulating signaling pathways and key gene expression (11). There are significant overlaps in the downstream targeted genes and molecules between ncRNAs and HBx, leading to a crossover in the carcinogenic mechanism (12, 13). These intersections may be key points in the interaction between HBx and ncRNAs and are likely to be the focus of future research.

## The interaction between ncRNA and HBV regulates the occurrence and development of HCC

Previous research on the molecular mechanisms of HCC has focused mainly on changes in protein levels; whereas, RNA has been considered a transient template in the protein synthesis process (14). However, with the continued discovery of a variety of ncRNAs that do not encode proteins, several ncRNAs have been found to participate in all aspects of cell life activities and play an important regulatory role in cancer (15). In fact, mRNAs encoding protein only account for 2% of the human genome, while ncRNAs are the majority (16). Abnormal expression of ncRNAs is common in carcinogenesis and is regulated and induced by the occurrence and development of HBV-related HCC (12). NcRNAs can be divided into long-chain ncRNAs (lncRNA, with a base number >200) and short-chain ncRNAs (with a base number is <200) based on their length (17). LncRNAs can be further divided into circular and non-circular lncRNAs depending on their structure (18). Among them, circular RNA (circRNA) also participates in the regulation of many processes in the onset and development of HBV-related HCC, such as the regulation of multiple key signaling pathways (19), or processes affecting liver inflammatory fibrosis and the immunosuppressive microenvironment (20). Normal lncRNAs also participate in a variety of carcinogenic mechanisms in HBV-related HCC, such as chromatin modification (21), regulation of transcriptional expression by transcription factors (22), modulation of protein stability (23), epigenetic silencing (24), mRNA splicing, and HBx-mediated lncRNA aberration (25). CircRNA, as an lncRNA with a circular structure, plays a unique role in HBV-related HCC due to its stable circular structure. It influences HCC proliferation, invasion, and metastasis by regulating multiple signaling pathways and targeted genes (26). Short chain ncRNA mainly include microRNA (miRNA), small nucleolar RNA (snoRNA), and PIWI interaction RNA (piRNA). MiRNAs primarily regulate gene expression at the transcriptional and post-transcriptional level (27), and usually lead to gene silencing through translation inhibition or catalytic cleavage of targeted mRNA by combining with complementary sequences (28, 29). There are differences in normal miRNA expression patterns in many cancers. For example, miR-506 has been implicated as a putative tumor suppressor in gastric cancer, exerting its inhibitory role by specifically targeting ZEB2 (zinc finger 2), thus contributing to suppression of tumorigenic processes (30). MiR-23a facilitates pancreatic cancer oncogenesis and progression by down-regulating the expression of the tissue factor pathway inhibitor (TFPI)-2 (31). In the past decade, many studies have shown that the HBx protein can promote the progression and metastasis of HCC by regulating miRNA expression (32). SnoRNAs can guide the chemical modification of rRNAs, tRNAs, and small nuclear RNAs (snRNAs) to enhance RNA folding. SnoRNAs are divided into two types, C/D box snoRNAs and H/ACA box snoRNAs, which regulate, respectively, methylation and pseudouridine of RNA (33). In the development of HBV-related HCC, different snoRNAs play different regulatory roles. For example, SNORD31 is down-regulated in HCC, and its decreased expression shows significant correlations with a larger tumor diameter, a high prevalence of vascular tumor embolism, extensive capsular infiltration, and poor tumor differentiation (34). SNORD17 exerts its inhibitory effect on the progression of HCC by suppressing the p53 signaling cascade. Simultaneously, p300 regulates p53 acetylation, facilitating its binding to the promoter region of SNORD17 and consequently repressing the expression of SNORD17 (35). piRNAs are an important class of sncRNAs that may be dysregulated during the occurrence and development of HCC. Some studies have found that these piRNA disorders are related to key signaling pathways during the onset and development of HCC (36). Therefore, ncRNAs play an important regulatory role in tumorigenesis through a variety of mechanisms. The role of different types of ncRNA in HBV carcinogenesis presents a network, interaction, and interdependence. However, current research on the interaction between ncRNA and HBV is still limited, which is also the focus and indicates the implicit difficulty of future research.

## miRNA and HBx regulation of HBV-related HCC carcinogenesis

MiRNAs play a key role in regulating gene expression during hepatocarcinogenesis (37). This regulation occurs primarily at the post-transcriptional level. MiR-122 is highly expressed tissue-specific miRNA in liver cells, accounting for 70% of total miRNA expression in HCC tissue (38). In chronic HBV infection, IL-6 and TNFa can inhibit miR-122 expression, which then up-regulates CCL2. MiR-122 directly inhibits the transcription factor *C/EBPα* and its own expression, by indirectly up-regulating the expression of the inhibitory transcription factor *C/EBPα* and *c-myc.* Inhibition of miR-122 expression during chronic hepatitis B (CHB) development in HCC promotes tumorigenesis (39). MiRNAs can regulate the expression of multiple genes and influence HBV proliferation, and are also regulated by the HBx protein, which in turn affects cell proliferation, cell metabolism regulation, immunity, methylation, apoptosis, epithelial-mesenchymal transformation (EMT), invasion, and metastasis of HCC. The HBx protein is an important carcinogenic protein encoded by HBV and plays a vital role in the occurrence and development of HCC by regulating multiple key signal pathways, affecting epigenetic expression, and promoting liver inflammation and fibrosis (40). Several studies have established a close relationship between the HBx protein and miRNA. HBx can regulate a variety of miRNAs that promote the occurrence and development of HCC. For example, the let-7a miRNA in the *let-7* family deregulated by HBx, targets the STAT3 signal pathway and *c-myc* to promote cell proliferation (41). HBx can also regulate lipid formation and promote HCC cell proliferation through the HBx/miR-429/*Rab18* pathway. Furthermore, HBx is positively correlated with miR-429 expression, while miR-429 can directly target *Rab18* to cause a disorder of lipid formation. Therefore, HBx can affect liver lipid metabolism and promote HCC cell proliferation by acting on *Rab18* through miR-429 (42). For the regulation of lipid formation and proliferation of hepatoma cells, HBx can also cause overexpression of acetyl-CoA synthetase long chain family member1 (*ACSL1*), which inhibits miR-205 expression and results in abnormal lipid metabolism. Targeting the 3’UTR causes down-regulation of *ACSL1* expression and cholesterol accumulation in hepatoma cells, which results in abnormal lipid metabolism (43). By negatively regulating the expression of miR-101, the HBx protein targets DNA methyltransferase 3A (DNMT3A), causing abnormal DNA methylation of the tumor suppressor gene, and promoting the appearance and development of HBV-related HCC (44). The HBx protein down-regulates miR-145, which reduces the inhibition of CUL5, promoting cell proliferation and the occurrence and development of HCC (45, 46). In HBV-related HCC, miR-132 plays a role in tumor inhibition through the AKT signaling pathway, while HBx can inhibit its transcription by methylation of the enhancer in miR-132 to promote the occurrence and development of HCC (47). Furthermore, miR-132 can regulate innate antiviral immunity by inhibiting the expression of the transcription co-activator *p300*. Whether HBx affects this critical innate immune regulation needs further study (48). In HBV-related HCC, HBx has been shown to suppress the expression of IL-12, while elevated expression of miRNA-21 significantly inhibits IL-12 expression and cellular apoptosis. Multiple cellular experiments indicate that HBx can induce miRNA-21 to suppress both cellular apoptosis and IL-12 expression (49). HBx inhibits *p53*-mediated activation of miR-148a, whose expression is downregulated in HBV-related HCC patients and exhibits a negative correlation with Hematopoietic pre-B cell leukemia transcription factor-interacting protein(HPIP). Conversely, HPIP is upregulated in HCC. Through the inhibition of HPIP-mediated mTOR signaling, miR-148a reduces the EMT, invasion, and metastasis of HCC (50). Finally, miR-520b can target the 3’UTR of HBXIP to inhibit its expression, while HBx can act on the complex formed by the transcription factor enhancer *sp1* and survivin upstream of miR-520b to inhibit miR-520b transcription, promoting the expression of HBXIP and the proliferation of HCC cells (51). In summary, previous studies have confirmed that HBx can directly or indirectly regulate miRNA to exert its effects in HBV-related HCC, this includes direct impact on miRNA activation and indirect influence on downstream protein expression of miRNA, involving participation in miRNA methylation modifications, thus influencing HCC. In future studies, the impact of HBx on upstream regulatory mechanisms of miRNA and the expression of downstream proteins will be pivotal areas of focus. But the mechanisms involved have not been fully understood; In addition, whether miRNA targets HBx and affects the carcinogenic process is also a blind spot, and it is worthwhile to further explore the mechanism of carcinogenesis induced by the interaction between HBx and miRNAs in the future.

## LncRNA and HBx regulation of HBV-related HCC carcinogenesis

LncRNA is commonly defined as a ncRNA that is longer than 200 bases. Compared to mRNAs, lncRNAs have fewer exons and a lower expression level (52). Although lncRNAs were considered to be functionally insignificant, with further studies, these have been found to play an important role in regulating carcinogenesis in various tumors, including HBV-related HCC. In HBV-related HCC, dysfunctional lncRNAs can regulate tumor proliferation, invasion, and metastasis (25). These lncRNAs can affect the development and prognosis of HCC through various mechanisms, including epigenetic changes, transcriptional activation, variable regulation of splicing, acting as competing endogenous RNAs (ceRNAs), regulating protein stability, and acting as precursors to miRNAs (53). HBx was also found to be involved in regulatory networks of some functional lncRNAs in the carcinogenic process. For example, the lncRNA *HULC* is positively correlated with HBx in HCC tissue samples. HBx activates the *HULC* enhancer through the cAMP response element binding protein (CREB), thus up-regulating the expression of HULC. In turn, *HULC* can promote cell proliferation by inhibiting the expression of the tumor suppressor gene *P18* located on the same chromatin (54). Furthermore, lncRNA HULC can promote the expression of the STAT3 transcription factor in the miR-539 transcription enhancer region; whereas, the HBx protein can enhance STAT3 expression and promote miR-21 expression. Experimental evidence demonstrated that *HULC* can stimulate miR-539 enhancer expression by activating HBx/STAT3 signaling, thus inhibiting APOBEC3B expression, reducing HBV cccDNA degradation and promoting HBV replication (55). HBx also regulates the transcription of lncRNA HOTAIR. Specifically, HBx regulates lncRNA HOTAIR transcription by regulating two transcription-inhibitory complexes, Polycomb Repressive Complex 2 (PRC2) and LSD1/Co-REST/HDAC1. Mitotic polo-like kinase 1 (Plk1) is activated by HBx and then induces the proteasome to degrade SUZ12 (an essential subunit of PRC2) and ZNF198 (a protein that can stabilize the LSD1/Co-REST/HDAC1 complex). Plk1 leads to histone modification and chromatin modification and ultimately results in activation of *HOTAIR* transcription. In HBV-related HCC, the down-regulation of SUZ12 and ZNF198 expression can also cause the epigenetic recoding of infected hepatocytes, thereby promoting the infiltration and metastasis of HCC (56). HBx can promote the hepatocarcinogenesis process through the HBx-lncRNA UCA1/EZH2-p27Kip1 axis. The expression of lncRNA UCA1 is positively correlated with HBx. HBx up-regulates the expression of lncRNA UCA1 and is associated with *EZH2* (the enhancer of histidine homolog 2, a histone methyltransferase), a component of the PRC2 chromatin modification complex PRC2. *EZH2* can inhibit p27Kip1 (a member of the CDKI protein family) by histone methylation of the p27Kip1 promoter. Silencing of *p27* improves the expression of CDK2 in cyclin-dependent kinase (CDK), which plays a crucial role in the G1/S phase transition. Consequently, *UCA1* can accelerate cell cycle progression in HBx-related HCC, enhance cell proliferation, and reduce cell apoptosis (57). Clinical data indicate that the expression of *DBH-AS1* is positively correlated with HBsAg. qRT-PCR revealed that the transcription of *DBH-AS1* was positively correlated with the expression of HBx mRNA, and *DBH-AS1* can activate the ERK/P38/JNK MAPK signaling pathway to stimulate the transition of G1/S and G2/M. The expression of *DBH-AS1* can also be significantly down-regulated by the tumor suppressor gene *TP53*. Therefore, *DBH-AS1* can serve as an HCC carcinogen gene (58). The lncRNA HBx-LINE1 pathway can activate Wnt/β-catenin signals to promote the occurrence and development of HCC; β-catenin can form a membrane-like complex with E-cadherin, acting as a cell adhesion protein and influencing the progression of EMT (59). Through the Wnt/β-Catenin signaling pathway, lncRNA HBx-LINE1 can simultaneously regulate the expression of c-myc, cyclinD1, and ZEB1, promoting HCC proliferation, infiltration, and metastasis (60). HBx can also significantly down-regulate the expression of lncRNA Dreh, which plays an inhibitory role in HBV-related HCC processes. HBx primarily achieves the downregulation of lncRNA Dreh by suppressing the expression of *Dreh.* LncRNA Dreh binds to intermediate filament vimentin and down-regulates its expression, which further changes the structure of the cytoskeleton to inhibit cell infiltration and migration (61). Furthermore, HBx can enhance the transcription of lnc01152, and lnc01152 can bind to the enhancer of the IL23 gene, an important inflammatory cytokine; thus, promoting its transcriptional activity and up-regulating STAT3 and p-STAT3, which promote the proliferation and tumorigenesis of HBV-related HCC cells (62). Furthermore, HBx can regulate autophagy in HBV-related HCC cells. HBx-induced autophagy can significantly increase the expression of lncRNA ATB and TGF-β, promoting cell infiltration and migration (63). Previous studies have shown the presence of a specific host gene for lncRNA, namely the lncRNA SNHG20, which is positively regulated by HBx. SNHG20 has the ability to reduce PTEN expression, thus promoting HCC proliferation through the SNHG20/PTEN pathway (64). A diverse range of lncRNA interact with the HBx protein in HBV-related HCC tumorigenesis, acting on different targets and exerting regulatory roles. LncRNA functions primarily by regulating multiple pathways, influencing gene expression, and modifying epigenetic mechanisms. Further research is required to elucidate how HBx affects host gene expression through modulation of lncRNAs, as well as the regulatory effects of lncRNAs on the HBx protein. Furthermore, the discovery of additional lncRNAs associated with HBx and the mechanisms of interaction with HBx are essential to better elucidate the regulatory mechanism underlying HBx-driven carcinogenesis. This will provide new possible targets for the prevention and treatment of HBV-related HCC.

## Interactions with other small ncRNA and HBx in HBV-related HCC carcinogenesis

In addition to the most common miRNAs, snoRNAs, and piRNAs also exert unique activity during HBV-related HCC carcinogenesis. Different snoRNAs play different roles in HCC. For example, the expression of SNORD113-1 is significantly decreased in HCC tissue and is closely associated with the worst prognosis of HCC. Functionally, SNORD113-1 can inactivate ERK1/2 and SMAD2/3 phosphorylation in the MAPK/ERK and TGF-β signaling pathway, respectively, thus playing an inhibitory role in HCC carcinogenesis. SNORD113-1 may become an important diagnostic and therapeutic target (65). *SNORD126*, encoded by the intron of cyclin B1 interacting protein 1 (CCNB1IP1), is highly expressed in HCC. Highly expressed *SNORD126* can promote cell proliferation and thus promote the development of HCC by regulating the PI3K/AKT pathway via activation of AKT phosphorylation and increasing fibroblast growth factor receptor 2 (FGFR2) expression (66). PiRNA also plays an important role in HCC. Law et al. discovered that PiR-Hep1 is significantly up-regulated in HCC tissue by deep sequencing of the miRNA transcriptome. It can influence AKT phosphorylation and activate the associated pathway to promote proliferation, thus accelerating the carcinogenic process of HCC (67). Although the interaction between other types of small ncRNA and HBx has not been thoroughly investigated, it is an important part of the regulation of HCC tumorigenesis, involving many key signaling pathways and targeted genes. In the future, strengthening research on the interaction between HBx and small ncRNA is a field worthy of further exploration.

## Interactions between circRNA and HBx and regulation of HBV-related HCC carcinogenesis

In recent years, circRNA, a type of ncRNA, has been found to play an important role in the development of many types of cancer, including HCC (68, 69). CircRNA is a unique type of ncRNA, characterized by a covalent closed circular structure without 5’-phosphate terminal or a 3’-hydroxy terminal (70). Its downstream 5’ splicing site is inversely connected with the upstream 3’ terminal (71). Functionally, circRNAs are rich in miRNA-binding sites, which allow them to act as a miRNA sponge and relieve miRNA-mediated inhibition of targeted genes, thus up-regulating their expression (72). CircRNAs can also participate in protein coding, influence the protein localization, and modulate protein function (73). The mechanism of HBx carcinogenesis involves the regulation of multiple signaling pathways through the regulation of different target molecules. YTHDC1, an N^6^ methyladenosine (m^6^A) reader, can promote reverse splicing of m^6^A-modified circRNA. RNA immunoprecipitation data reveals that exposure to the YTHDC1 antibody can enrich circARL3 levels, an effect that is significantly amplified by HBx expression. Thus, HBx can increase the expression of circARL3 through m^6^A modification, which in turn, sponges miR-1305 and up-regulates the expression of oncogenes such as *WNT2, UBE2T, MDM2, TGF-β2*, and *POLR3G*, leading to the development of HBV-related HCC (74). HBx also inhibits the formation of circSFMBT2 by binding to the lateral *ALU* element and interacting with DExH box helicase 9 (DHX9). This inhibition reduces sponge adsorption of miR-665 and then up-regulates the tissue metalloproteinase inhibitor 3 (TIMP3), promoting HCC infiltration and metastasis (75). Few investigations have investigated the interaction between HBx and circRNA, which remains the focus of research. For example, HBx can up-regulate the expression of β-catenin and its target genes, cyclin D1, and *c-myc* proto-oncogenes activating the COX-2/Wnt/β-catenin pathway through COX-2 to promote the proliferation of HL-7702 cells (76). Meanwhile, circRNAITCH is significantly down-regulated in HCC tissue and inhibits HCC cell proliferation by regulating the Wnt/β-catenin signal pathway and inhibiting the expression of *c-myc* and cyclin D1 (77). In the PI3K and AKT pathways, HBx is associated with miR-21 expression. HBx up-regulates the expression of PTEN through miR-21 to reduce the expression of PI3K and AKT, thus inhibiting the activity of MMP2 (78). Conversely, exosome-transported circMMP2 increases the expression of its host gene *MMP2* caused by the sponge adsorption of miR-136-5p. Rescue experiments have shown that miR-136-5p and *MMP2* play an important role in HCC metastasis (79). In the NF-κB pathway, HBx up-regulates the expression of the genes *VEGF* and *MMP* in HBV-related HCC, influencing the invasion and metastasis of HCC (80). circRNAs via NF-κB that regulate *VEGF* and *MMP* expression have also been a research hotspot. For example, in RNA pull-down assays and mass spectrometry detection, circLIFR overexpression has been shown to up-regulate several regulatory genes, including *VEGF*. circLIFR and TANK-binding kinase (TBK1) up-regulate the expression of *MMP13*, *MMP3*, and *VEGF*, which promote HCC proliferation, invasion, and metastasis (81). CircDLC1 interacts with *HUR* to inhibit MMP-1 expression in HCC, thus inhibiting HCC proliferation (82). Conversely, has-circ-0001806 up-regulates MMP16 expression by inhibiting miR-193a-5p, promoting the occurrence and development of HCC (83). These signal pathways play crucial roles in various metabolic and cellular activities. The interactions between several ncRNAs and HBx have been confirmed. The stable circular structure of circRNA highlights the importance of exploring its interaction with the HBx protein, which remains a focus and a challenge for future research. The extensive overlap between HBx and circRNA in the regulation of the pathogenesis and development of HCC involves the common role of miRNA and in the regulation of gene expression. Therefore, there are compelling reasons to believe in the existence of an interaction between circRNA and the HBx protein in the regulation of HCC pathogenesis. Investigating this interaction in greater depth will improve our understanding of the mechanisms underlying the occurrence and development of HCC and will provide new insight for future research.

In summary, interactions between HBx protein and ncRNA play a significant role in the pathogenesis of HBV-related HCC. HBx regulates a variety of ncRNAs to influence the expression of downstream molecular targets, as summarized in **Table 1**. However, there is still a lack of relevant research on the regulatory relationship between HBx and circRNAs. Based on existing research, multiple signaling pathways and target genes are implicated in HBx-mediated carcinogenesis. HBx regulates the transcription and expression of lncRNAs in various ways. Because of its stable circular structure, circRNA degradation is reduced, and the cross-regulation between molecules regulated by circRNAs and by HBx is enhanced. At the same time, circRNA sponges miRNAs that may interact with HBx; thus, the interaction between circRNAs and HBx may have a more significant impact on the occurrence and development of HBV-related HCC. In the future, studies evaluating the interaction between ncRNAs and the HBx protein in HCC will help us to further reveal the carcinogenic mechanism of HBV *in vivo* and will also provide a new basis for future research.

**Table 1:** Target and function of the interaction between HBx and ncRNA.

ncRNA	HBX-ncRNA target	HBX-HCC target(s)	Mechanism	Functions	References
miR-429	/	*Rab18*	Deregulation	Lipid metabolism, cell proliferation	(42)
miR-205	/	3’UTR/*ACSL1*	Deregulation	Lipid metabolism	(43)
miRNA-21	/	IL-12	Upregulation	Cell apoptosis	(49)
miRNA-148a	*P53*	HPIP	Deregulation	EMT,invasion,metastasis	(50)
miR-520b	Sp1-survivin	*HBXIP*	Deregulation	Cell Proliferation	(51)
lncRNA HOTAIR	PIK1/PRC2/ZNF198	/	Up-regulation	Epigenetic reprogrammed, cell metastasis	(56)
lncRNA UCA1	EZH2	CDK2	Up-regulation	Cell proliferation, apoptosis	(57)
lncRNA HULC	CREB	*P18*	Up-regulation	Cell proliferation	(54)
lncRNA HULC	*STAT3*	*APOBEC3B*	Up-regulation	Decrease HBV cccDNA degradation, cell proliferation	(55)
lncRNA DBH-AS1	/	ERK/P38/JNK MAPK	Up-regulation	Oncogene, cell proliferation	(58)
lncRNA HBx-LINE1	/	*myc/cyclinD1/ZEB1*	Up-regulation	EMT, cell proliferation, metastasis, invasion	(60)
lncRNA SNHG20	/	*PTEN*	Up-regulation	Cell proliferation	(64)
circARL3	m^6^A	*WNT2/UBE2T/MDM2/TGF-β2/POLR3G*	Up-regulation	Oncogene	(74)
circSFMBT2	DHX9	TIMP3	Deregulation	Cell metastasis, invasion	(75)

"/":The mechanisms and molecules involved in the current research have not yet been elucidated, and further research is needed.

## Conclusions

**Figure 1:**
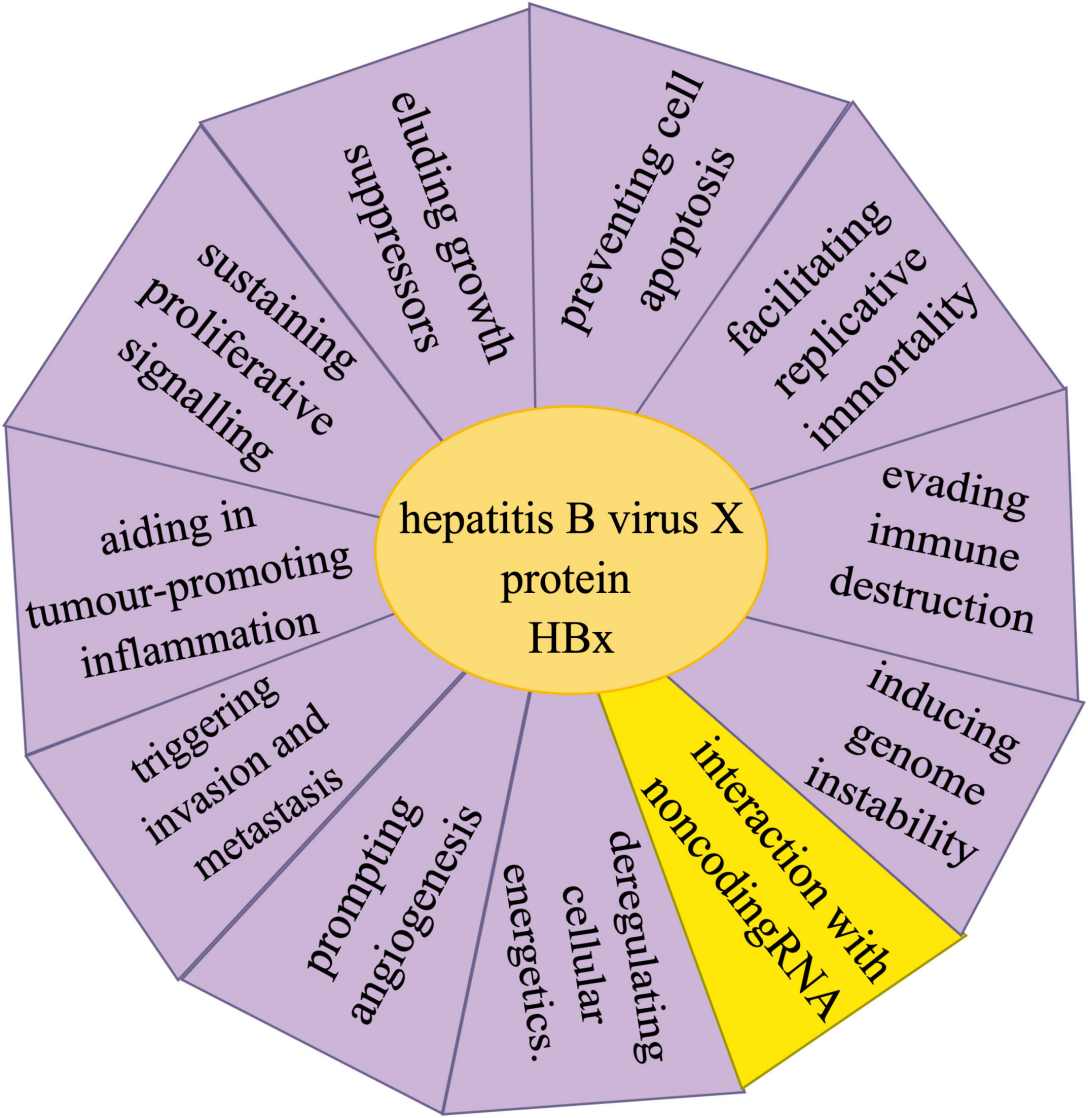
Hallmarks involved in the hepatitis B virus X protein (HBx) carcinogenic process of hepatitis B virus-related hepatocellular carcinoma include sustaining proliferative signaling, eluding growth suppressors, evading immune destruction, facilitating replicative immortality, aiding in tumor-promoting inflammation, triggering invasion and metastasis, prompting angiogenesis, inducing genome instability, preventing cell apoptosis, and deregulating cellular energetics, as well as the interactions between HBx and non-coding RNA.

HBV-related HCC is a type of malignant cancer that poses a threat to human life and health, and is responsible for a high proportion of cancer cases. In China, although the prevalence of HBV is decreasing due to improved health conditions, it remains the main cause of HCC. Consequently, research on the diagnosis, treatment, and prognosis of HBV-associated HCC continues unabated. NcRNAs, which do not encode proteins, were previously overlooked. However, with continued technological advancements, ncRNAs have now been recognized to play an essential regulatory role in the occurrence and development of HBV-related HCC. NcRNAs influence the activities of multiple key signaling pathways, act on tumor suppressor and oncogenic genes, and are involved in the formation of liver inflammatory fibrosis, and an immunosuppressive microenvironment. CircRNAs, with their stability derived from its unique structure, ability to sponge adsorption of miRNAs, and their complex interaction with proteins, play a significant role in various diseases, including HCC. Relevant research on HCC has led to many new discoveries that are critical for diagnosis, treatment, and prognosis of HCC. Several mechanisms are involved in the carcinogenic process of HBx, among which the interaction between HBx and ncRNAs is closely related (**Figure 1**). Currently, research has mainly focused on the regulation and expression of multiple ncRNA by HBx. However, limited research has been conducted on the role of endogenous ncRNAs in HBx-induced carcinogenesis. Furthermore, there is a lack of research on the interaction between HBx and circRNAs. Therefore, future studies should focus on the interaction between ncRNA and HBx carcinogenesis, which will provide new insights into the mechanisms of HBV carcinogenesis and on the prevention and treatment of the carcinogenetic process after HBV infection. More in-depth research is required to identify key molecules and mechanisms.

## Author contributions

Concept, design, and supervision, JY, NL, GC, and ZW. Material support, ZW and PC. Drafting of the manuscript, ZW, CZ, and NL. Obtained funding, JY. Critical revision of the manuscript for important intellectual content, all authors. All authors read and approved the final version of the manuscript.
